# Climate Effects on High Latitude *Daphnia* via Food Quality and Thresholds

**DOI:** 10.1371/journal.pone.0126231

**Published:** 2015-05-13

**Authors:** Anna Przytulska, Maciej Bartosiewicz, Milla Rautio, France Dufresne, Warwick F. Vincent

**Affiliations:** 1 Centre d’études nordiques (CEN), Université Laval, Québec, Québec, Canada; 2 Département de biologie, Université Laval, Québec, Québec, Canada; 3 Centre Eau Terre Environnement, Institut National de la Recherche Scientifique, Québec, Québec, Canada; 4 Department of Fundamental Sciences, Université du Québec à Chicoutimi, Chicoutimi, Québec, Canada; 5 Biology, Chemistry and Geography Department, Université du Québec à Rimouski, Rimouski, Québec, Canada; University of Connecticut, UNITED STATES

## Abstract

Climate change is proceeding rapidly at high northern latitudes and may have a variety of direct and indirect effects on aquatic food webs. One predicted effect is the potential shift in phytoplankton community structure towards increased cyanobacterial abundance. Given that cyanobacteria are known to be a nutritionally poor food source, we hypothesized that such a shift would reduce the efficiency of feeding and growth of northern zooplankton. To test this hypothesis, we first isolated a clone of *Daphnia pulex* from a permafrost thaw pond in subarctic Québec, and confirmed that it was triploid but otherwise genetically similar to a diploid, reference clone of the same species isolated from a freshwater pond in southern Québec. We used a controlled flow-through system to investigate the direct effect of temperature and indirect effect of subarctic picocyanobacteria (*Synechococcus*) on threshold food concentrations and growth rate of the high latitude clone. We also compared the direct effect of temperature on both *Daphnia* clones feeding on eukaryotic picoplankton (*Nannochloropsis*). The high latitude clone had a significantly lower food threshold for growth than the temperate clone at both 18 and 26°C, implying adaptation to lower food availability even under warmer conditions. Polyunsaturated fatty acids were present in the picoeukaryote but not the cyanobacterium, confirming the large difference in food quality. The food threshold for growth of the high latitude *Daphnia* was 3.7 (18°C) to 4.2 (26°C) times higher when fed *Synechococcus* versus *Nannochloropsis*, and there was also a significant negative effect of increased temperature and cyanobacterial food on zooplankton fatty acid content and composition. The combined effect of temperature and food quality on the performance of the high latitude *Daphnia* was greater than their effects added separately, further indicating the potentially strong indirect effects of climate warming on aquatic food web processes.

## Introduction

The Arctic is currently warming at much faster rates than the global average and many physical effects including reduced seasonal ice cover over lakes and seas, a deepening of the permafrost active layer, and changes in snowfall and hydrology, have become apparent in northern environments [1]. High latitude lakes have been identified as systems that are particularly vulnerable to warming because of the wide-ranging influence of low temperatures and persistent ice cover on their ecosystem structure and function [2]. The effects of loss of ice cover on aquatic food webs and productivity have been investigated via field observations, simulated changes in underwater irradiance, and by paleolimnological inferences [3,4,5]. Similarly, temperature effects have been examined by observation, experimental manipulations and modeling [6,7,8]. Collectively, these studies imply that climate change has the potential to directly affect aquatic communities and processes through changes in light and temperature conditions, but may also exert indirect effects via changes in species composition and trophic relationships. However, despite increasing interest in climate impacts on high latitude ecosystems, the combined influences of such direct and indirect effects on trophic processes, and specifically phytoplankton–zooplankton interactions, remain poorly understood.

Picocyanobacteria are a ubiquitous component of the phytoplankton in high latitude freshwaters [9], and may be increasingly favored by climate warming. At temperate latitudes, cyanobacterial growth is known to respond strongly to warmer temperatures and increased nutrient supply [10], and high latitude cyanobacteria may be similarly responsive. For example, loss of ice from a High Arctic lake resulted in increased mixing and nutrient entrainment from lower depths, and these conditions were accompanied by a 5-fold increase in picocyanobacterial concentrations [11]. In experimental analyses of temperature and UVR effects on microbial communities in Arctic lakes, warmer temperatures resulted in a more rapid net growth (chlorophyll *a*) of smaller (< 2 μm) than larger phytoplankton, with noticeable dominance of picocyanobacteria [12]. More recent studies have indicated that warming may not only stimulate some cyanobacterial species in cold benthic environments, but may also lead to lower cyanobacterial diversity and increased toxin production [13].

An increased prevalence of cyanobacteria and the associated shift towards a less diverse phytoplankton community could potentially affect food web processes in northern waters given that many of these prokaryotic taxa are known to be a poor food source for zooplankton grazers. For filamentous and colonial cyanobacteria this is in part because their morphological traits interfere with ingestion [14], but single celled picocyanobacteria may also be a less favorable food supply than other phytoplankton. A diet consisting of cyanobacterial cells was shown to lead to decreased growth rates and higher threshold food concentrations in temperate zooplankton [14], likely due to decreased food quality. The latter includes low phosphorus to carbon ratios [15] and the absence of polyunsaturated fatty acids [16].

Zooplankton in high latitude lakes and ponds are exposed to a variety of environmental stresses ranging from extreme UVR and low water levels to prolonged periods of low temperatures and food shortages [17]. An interesting feature of northern crustaceans is the trend towards polyploidy at high latitudes [18,19], which may be accompanied by increased cell and body size [20]. The larger body sizes in zooplankton, and particularly in cladocerans, may result in competitive superiority due to lower threshold food concentrations [21]. Cladocerans are often the dominant constituents of the zooplankton communities and keystone herbivores in high latitude ponds [17], and polyploidy is well known in northern taxa in this group [17], including in the genus *Daphnia* [22].

Our aim in the present study was to evaluate the direct effect of temperature on zooplankton feeding, and indirect effects that may operate in high latitude freshwaters through a shift of the phytoplankton community to increased dominance by picocyanobacteria. We hypothesized that high latitude cladocerans will respond negatively to picocyanobacteria as a food source. Given evidence that high latitude *Daphnia* populations are adapted to colder temperatures [22,23], we also hypothesized that subarctic *Daphnia pulex* will be negatively affected by warming to a greater extent than its southern counterparts. To address these hypotheses, we compared the effects of temperature on food thresholds and growth rates of a high latitude versus temperate clone of *Daphnia pulex*, and the effects of picoplankton food type (picoeukaryote versus picocyanobacterium) on the high latitude clone, including its fatty acid content and composition.

## Material and Methods

**Fig 1:**
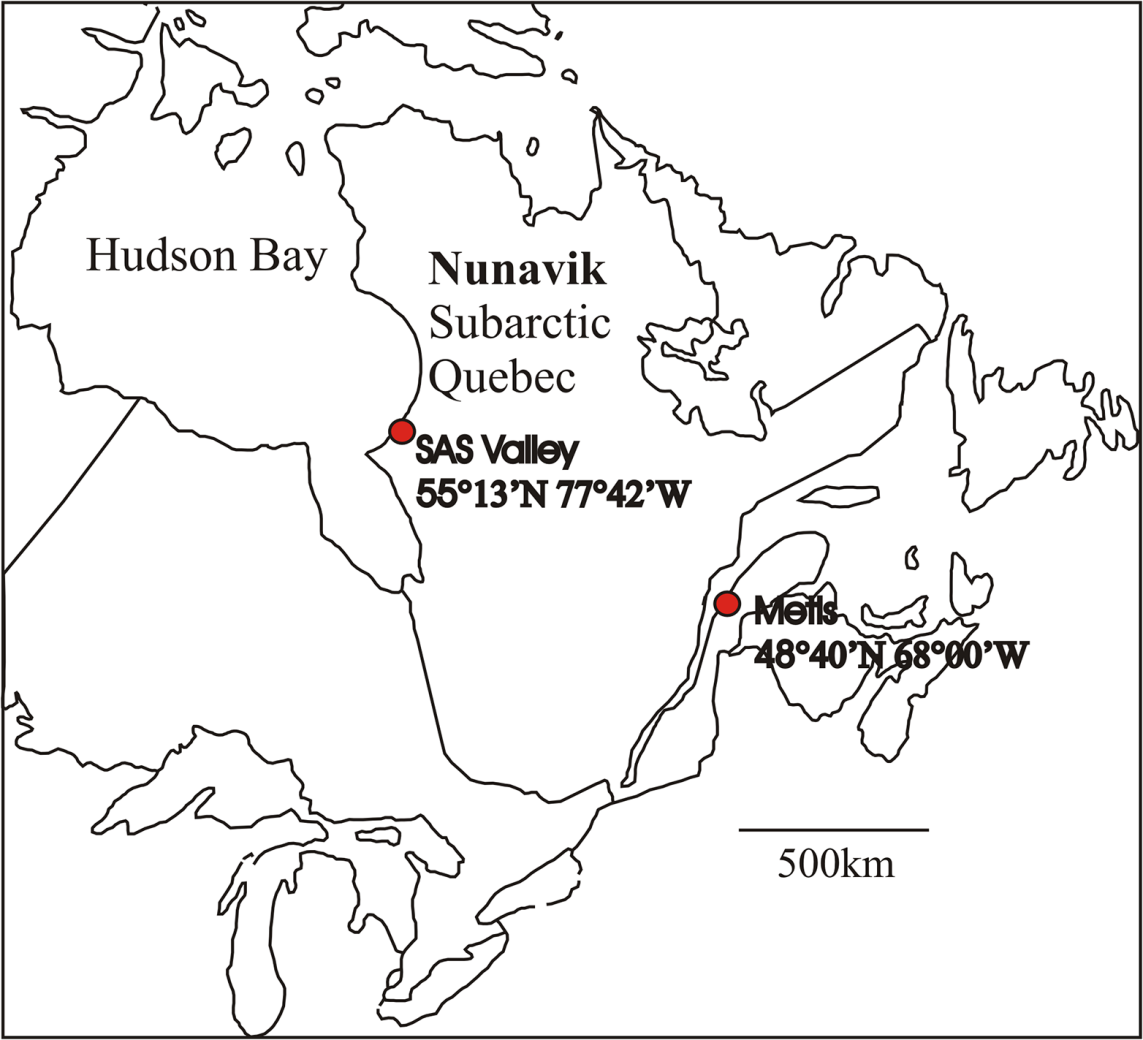
Location of the collection site for the zooplankton used in the growth experiments. The high latitude clone of *D*. *pulex* (*Daphnia*-HL) originated from a thermokarst pond in the SAS Valley (subarctic Québec) and the temperate clone from Metis (Jardins de Métis) in southern Québec (*Daphnia*-T).

We isolated a high latitude clone of *Daphnia pulex* (*Daphnia-*HL) from a permafrost thaw lake (SAS2C; 55°13’N, 77°42’W) in the Sasapimakwananisikw River Valley (SAS Valley) located near the village of Kuujjuarapik-Whapmagoostui at the edge of Hudson Bay, subarctic Québec, Canada (Fig 1). No collection permits were required, but consultation about this work was undertaken with the Whapmagoostui First Nation and the Kuujjuarapik Inuit community via Centre d'études nordiques (CEN). The *Daphnia-*HL clone was maintained in 0.45 μm filtered temperate lake water (Lake Saint Charles, Québec, Canada), supplemented with selenium (7 μg SeO_2_ L^-1^), at room temperature (21°C) over 12 months prior to the start of the experiments. There was unlikely to be any genetic change in the *Daphnia* population within this time frame since no sexual reproduction occurred and asexually produced ephippia were removed. The temperate clone of *Daphnia pulex* (*Daphnia-*T) was isolated from a shallow temperate pond in the Jardins de Métis, southern Québec, Canada (48°37’N, 68°06’W) and was maintained in the same laboratory conditions as the high latitude clone. This “common garden” approach allowed direct comparisons between the two genotypes with the same starting conditions. The two pond sites are 980 km and 6 degrees of latitude apart, and this translates into a large climatic difference. For the high latitude clone, the duration of the growing (open water) season would likely be 4 months (June to September), with 12–13°C as an average surface water temperature at the beginning of the season rising to 16–17°C by mid-summer [24], and extremes to around 20°C [25]. For the temperate clone, the average growing season would likely be 6 months (May to October) with 12–13°C as an average surface water temperature at the beginning and by the end of the season and 22–24°C in the middle of the summer.

### Genetic analyses

In order to assign the two *Daphnia* clones to their respective lineages in the *Daphnia pulex* complex, we sequenced the NADH dehydrogenase subunit 5 (ND5) mitochondrial gene. DNA extractions were performed using 40 μL of Quick extract (Epicentre Biotechnologies) solution. Individual *Daphnia* were incubated for 2.5 hours at 65°C followed by 15 minutes at 95°C. The ND5 gene was amplified using the primers DpuND5b (5**′**-GGGGTGTATCTATTAATTCG-3**′**) as reverse primer and DpuND5a (5**′**-ATAAAACTCCAATCAACCTTG-3**′**) as in [26]. DNA sequencing was done by the Genome Québec laboratory at McGill University. DNA sequences were aligned using MUSCLE [27] and a maximum-likelihood phylogeny was produced using phyML 3.0 [28] to identify the position of both clones in the ND5 phylogeny and ascertain their mitochondrial origin. Sequences from a number of clones belonging to the major lineages of the *D*. *pulex* complex (available in GenBank) were added to the tree.

The genome size of both clones was assessed using flow cytometry with *Artemia franciscana* as a standard. Two *Daphnia* were ground using a Kontes Dounce tissue grinder for 20 strokes with an ‘A’ pestle in 1 mL of modified Galbraith buffer [29]. After filtering twice through a 40-μm mesh, 20uL of cell suspension of *Artemia franciscana* were added to each sample. Cell suspensions were stained with propidium iodide (50 ppm; Invitrogen) at 4°C in the dark for 10 h. The nuclear DNA content of each clone was assessed using an Epics Altra flow cytometer (Beckman-Coulter) with an argon laser emitting 14 mW of light at 488 nm; DNA-PI fluorescence emission was measured at 600–640 nm. Instrument alignment and stability were monitored by adding 5 μL of a solution of red-fluorescing beads (Linear Flow Carmine; Molecular Probes). Nuclear DNA content was calculated from the mean fluorescence intensity (FL, arbitrary units) on the gated data of the first peak as: Total genome size = (sample FL/ *Artemia* FL) x 2.61 pg where 2.61 pg corresponds to the diploid genome size of *Artemia franciscana*.

### Growth experiments

Juveniles of the high latitude *Daphnia pulex* (*Daphnia-*HL) clone were exposed to seven abundances of either picoeukaryotic (*Nannochloropsis limnetica*, strain 18.99, SAG culture collection; Eustigmatophyceae) or picocyanobacterial (subarctic *Synechococcus* sp., strain PCCC-211, Université Laval; Chroococcales) food, at either 18°C or 26°C. The selection of food levels was based on two preliminary experiments indicating that animals did not survive at elevated temperature in food concentrations below 0.01 mg C L^-1^ and that they produced eggs before the end of the experiment in food concentrations higher than 0.2 mg C L^-1^. Following the experiments using *Daphnia-*HL, two additional experiments (*Nannochloropsis* only, at 18 and 26°C) were conducted in the same experimental system using the temperate clone of *Daphnia pulex* (*Daphnia-*T). All of the experiments were performed in a set of flow-through vessels [30] installed in a water-bath with the temperature controlled to within ±0.5°C. The phytoplankton cell abundance was maintained at the required food carbon levels with a multichannel peristaltic pump providing fresh medium from a magnetic stirred, covered, black PVC reservoir, at a rate of 2.2 L d^-1^ per chamber. The medium was prepared daily during each of the experiments in a two-step lake water filtration procedure (through 0.45 and 0.2 μm Advantec membrane filters) to remove all bacteria and detritus that could be a possible additional food source for *Daphnia* [31].

The neonates used in each experiment were derived from fourth brood *Daphnia* females cultured at 21°C in three 80 L tanks and fed daily *ad libitum* with equal mixture of picoeukaryotic cells and picocyanobacterial cells. Prior to each experiment, the neonates (0 to 8 hours old) were separated from females and placed for 2 days in 2.5 L glass beakers within a water-bath maintaining temperatures similar to those used during subsequent experiment, containing fresh experimental medium, but supplemented only with phytoplankton food that was later used in the experiment (1 mg C L^-1^ of either phytoplankton species).

The picophytoplankton were cultured in chemostats containing Z8 medium [32] and harvested daily for the zooplankton medium. The organic carbon content of these cell suspensions used in each experiment was calculated using a calibration curve relating organic carbon to absorbance at 800 nm.

Each of the experiments was started with 700, 2-d old (±8h) juveniles (25 animals per flow-through chamber) that were distributed at random into 28 flow-through vessels (4 replicates for each of the 7 food concentrations) and kept under experimental conditions for 4 days. At the same time 100, 2-d old, neonates were measured under a dissecting microscope (Zeiss) and freeze-dried for analysis of fatty acids (FA), separated by fraction: saturated fatty acids (SAFA), monounsaturated fatty acids (MUFA) and polyunsaturated fatty acids (PUFA), as in [33]. Additional animals were dried at 60°C for 12 h (10×10 individuals in a pre-weighed silver cups) for mass estimation using a Sartorius high precision balance. After the 4^th^ day, 10 random *Daphnia* from each flow-through vessel were harvested and dried in 60°C (12h) for mass estimation and the remaining 15 animals from each chamber were freeze dried in a 4 mL cryovial for fatty acid analysis. The growth rates were calculated from body dry mass as g = (ln M_t_—ln M_0_)/t where M_t_ is body mass of 6-d old individuals, and M_0_ represents the body mass of 2-d old individuals.

Threshold food concentrations (TFC) were calculated as the *x*-axis intercepts for linear regressions of growth rate versus natural log (*ln*) phytoplankton abundance [21]. The TFC and slopes of the regressions among treatments were tested for differences using 95% confidence intervals (CI): significant differences were identified by non-overlapping CIs. The TFC confidence interval for each treatment was estimated with the slope and y-axis intercept coefficients from the regression equations. A three-way ANOVA (food concentration, temperature and food type) followed by Tukey HSD was used to test for significant effects of experimental factors on the growth rate in *Daphnia-*HL in the first series of experiments and three-way ANOVA (food concentration, temperature and clone) was used to test for significant effects of temperature and differences in growth rate between the *Daphnia*-HL and *Daphnia-*T clones in the second series of experiments. The fatty acid content and composition (FA, total fatty acids; SAFA, MUFA and PUFA contents) were compared using two-way ANOVA (temperature, food type) followed by Tukey HSD, with measurements of animals from the 5 intermediate food abundances used as replicates. All statistical analyses were performed using JMP Software from SAS.

## Results

### Phytoplankton and zooplankton characteristics

**Fig 2:**
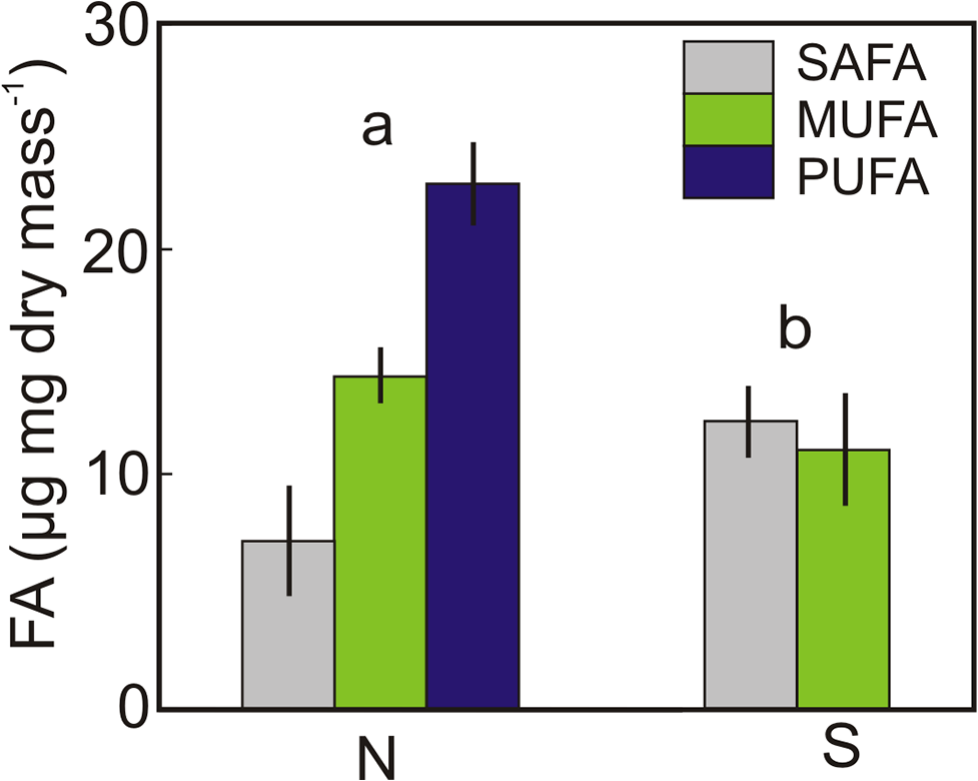
The FA content and composition of two picophytoplankton food types used in the growth experiments. The concentrations of saturated (SAFA), monounsaturated (MUFA) and polyunsaturated (PUFA) fatty acids (±SE, n = 5) in *Nannochloropsis* (N) and *Synechococcus* (S). The different letters indicate a significant difference in total fatty acid content at p<0.001.

The strain of *Nannochloropsis* used in this study contained almost twice the quantity of fatty acids (FA) per unit biomass as the subarctic *Synechococcus* (43.7 and 24.0 μg FA mg^-1^ dry mass^-1^, respectively), including PUFAs that were completely absent from the picocyanobacteria (Fig 2). The *Daphnia* neonates kept at 26°C for 2 d after birth were slightly heavier than those kept at 18°C, but the difference was significant only for animals at 26°C fed *ad libitum* with *Synechococcus* (mean = 11.7 μg, n = 10, *p* = 0.001). There was also a small but significant difference in body length between the two clones: 0.71 (±0.033) mm for *Daphnia*-HL versus 0.63 (±0.019) mm for *Daphnia*-T (t = 3.73, *p* = 0.03, df = 18).

Both *Daphnia* clones clustered in the *Daphnia pulex* lineage on the maximum-likelihood tree (S1 Fig) and showed a >99% similarity in their mitochondrial ND5 gene sequences. The high latitude clone had a genome size of 0.51 pg, whereas the temperate clone had a genome size of 0.34 pg. The ratio of these values (1.5) indicated that the high latitude *Daphnia* is a triploid clone while the temperate *Daphnia* clone is diploid.

### Growth rate and threshold food concentration responses

**Fig 3:**
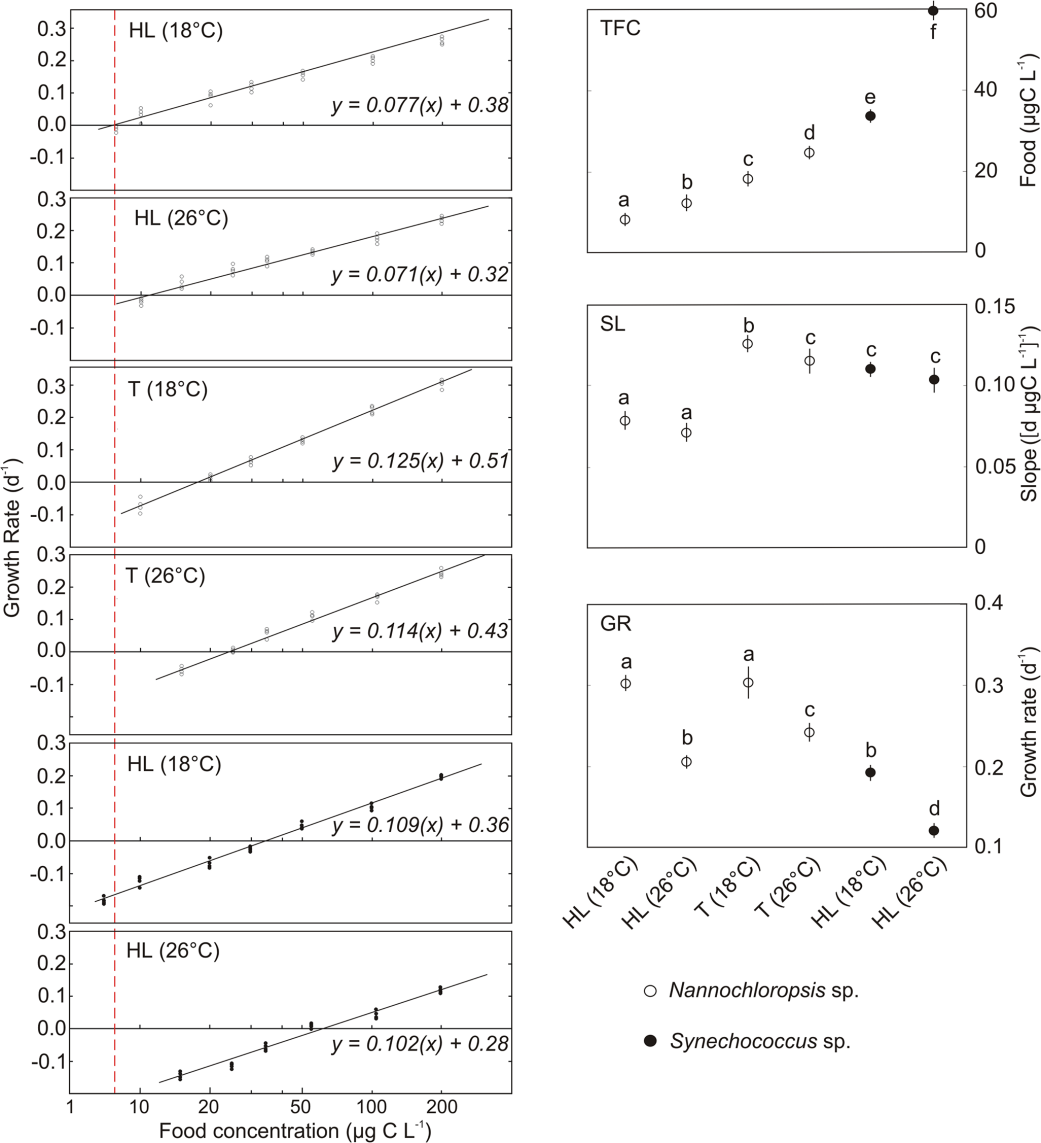
Growth versus food relationships. The left hand graphs show growth rates against *ln* food concentration in each experiment, with the fitted regression equations giving growth rate as a function of *ln* food concentration in mg C L^-1^; each point in the graphs is the mean of 4 replicates at each food concentration. The right hand graphs show threshold food concentrations (TFC, mean ± 95% CI), slopes of the regression lines of growth rate on food concentration (SL, mean ± 95% CI) and growth rates at 0.2 mg C L^-1^ (GR, mean ± 95% CI) in high latitude (HL) and temperate *Daphnia* (T). The animals were fed *Nannochloropsis* or *Synechococcus* (HL clone only) at 18°C or 26°C. The different letters indicate statistically different values (p<0.05, ANOVA with Tukey HSD or confidence intervals comparisons).

There was a significant effect of temperature and food type on the growth rates in *Daphnia*-HL (three-way ANOVA with Tukey HSD, Table 1, Fig 3) in the first series of experiments and a significant effect of experimental clone and temperature, as well as their interactions, on growth rates in the second series of experiments (three-way ANOVA with Tukey HSD, Fig 3). There was also a significant effect of temperature and clone on TFC in *Daphnia* (Fig 3), and a significant effect of temperature and food type on TFC in *Daphnia-*HL (Fig 3). The food threshold for growth in the *Daphnia-*HL and-T was higher at 26°C than at 18°C. The TFC was also significantly higher with *Synechococcus* than with *Nannochloropsis* food at both temperatures (Fig 3). When fed *Nannochloropsis*, the high latitude *Daphnia* was able to maintain positive growth at food abundances lower than the temperate clone at both temperatures (Fig 3).

**Table 1:** Effects on *Daphnia* growth rates.

Source	df	MS	F			p
***Daphnia*-HL**						
T	1	0.0371	440			**<0.0001**
F	1	0.4673	5547			**<0.0001**
T × F	1	0.0002	3			0.0931
FC	5	0.1216	1444			**<0.0001**
T × FC	5	0.0013	16			**<0.0001**
F × FC	5	0.0072	85			**<0.0001**
T× F × FC	5	0.0001	1			0.2754
Error	72	0.0001				
***Daphnia*-HL&T**						
T	1	0.0215	163			**<0.0001**
FC	5	0.1436	1088			**<0.0001**
T × FC	5	0.0019	15			**<0.0001**
Cn	1	0.0379	287			**<0.0001**
T × Cn	1	0.0009	7			**0.01**
FC × Cn	5	0.0128	97			**<0.0001**
T × FC × Cn	5	0.0003	2			**0.044**
Error	72	0.0001				

The first three-way ANOVA tested for the effects of temperature (T), food type (F), food concentration (FC) and their interactions on growth rate of *Daphnia*-HL, and the second tested the effects of temperature (T), clone (Cn), food concentration (FC) and their interactions on growth rate of the two *D*. *pulex* clones, *Daphnia*-HL and *Daphnia*-T.

The slope of the growth rate versus food abundance (*ln*) regression line was greater at 18 than 26°C, but this difference was significant only in *Daphnia-*T (based on CI). The slope of the regression was also significantly greater when *Daphnia-*HL were fed with *Synechococcus* than with *Nannochloropsis*. The slope for *Daphnia*-T was higher than for *Daphnia-*HL at both temperatures (Fig 3). The growth rates for both clones at the highest food concentration used in this study (0.2 mg C L^-1^) were significantly lower at 26 than 18°C, and lower for *Daphnia-*HL fed *Synechococcus* than *Nannochloropsis* (Fig 3). *Daphnia-*T fed *Nannochloropsis* (0.2 mg C L^-1^) grew faster than *Daphnia-*HL fed the same food, but only at 26°C (Fig 3).

### Fatty acid responses

**Fig 4:**
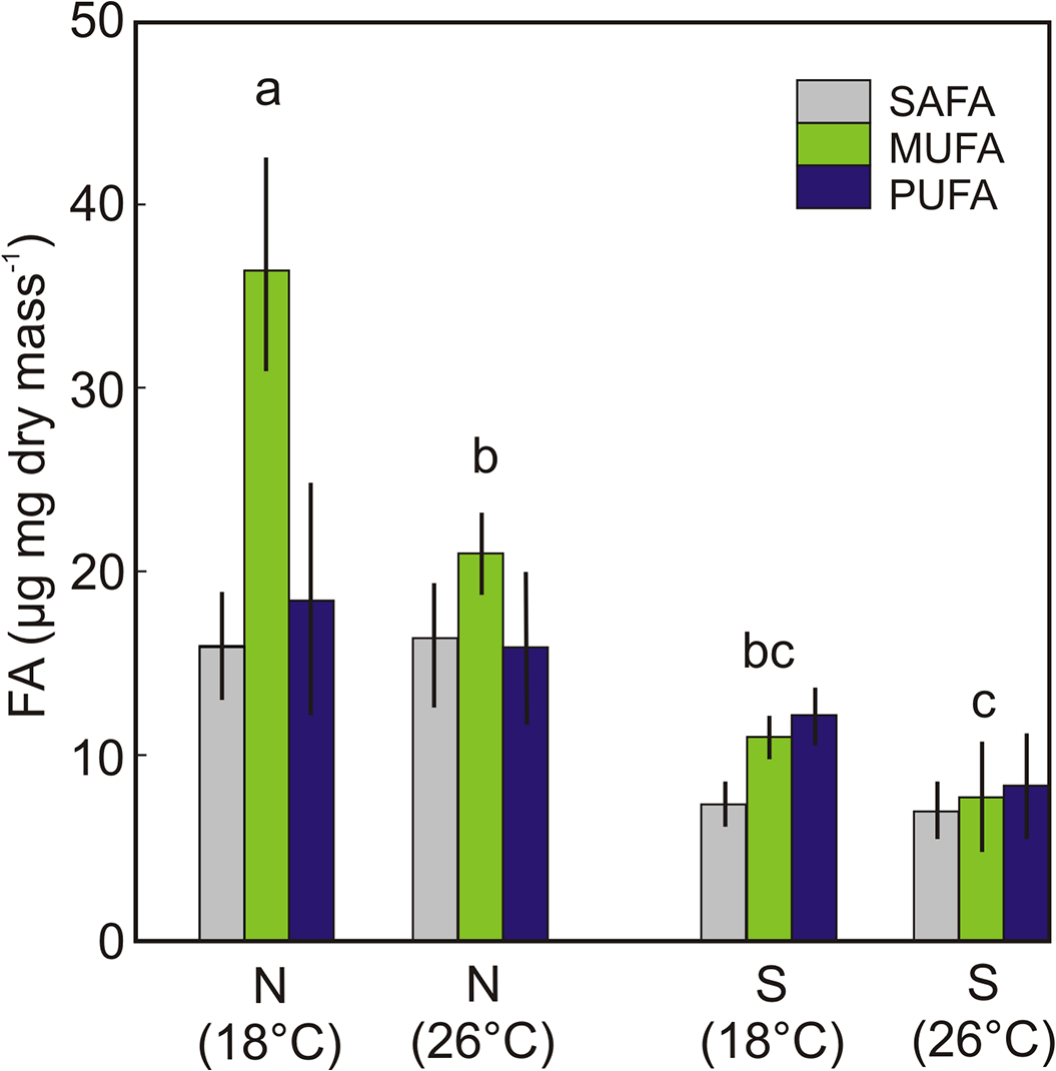
The content and composition of FA in *Daphnia*-HL fed either *Nannochloropsis* (N) or *Synechococcus* (S) at 18°C or 26°C. Each values is a mean of 5 replicates ±SE. Different letters indicate significant differences in total fatty acid contents at p<0.01 (two-way ANOVA with Tukey HSD).

The elevated temperature resulted in a significant decrease of body fatty acids (FA) in *Daphnia-*HL feeding on either *Nannochloropsis* or *Synechococcus* (Table 2, Fig 4). The animals feeding on *Nannochloropsis* accumulated more FA than those feeding on *Synechococcus* at each temperature (Fig 4). There was also a significant effect of the interaction of food and temperature on total body FA content (Table 2).

**Table 2:** Effects on *Daphnia* fatty acid content.

Source	df	MS		F	P
T	1	2690.7		24	**<0.001**
F	1	6114.3		55	**<0.0001**
F × T	1	913.7		8	**0.011**

The two-way ANOVA tested the effects of temperature (T), food type (F) and their interaction on total body fatty acids in *Daphnia-*HL.

The warmer temperature resulted in a significant difference in FA composition in *Daphnia-*HL (Fig 4). The MUFA content was higher in animals grown at 18 than 26°C, but this difference was significant only in the *Nannochloropsis* food treatment (*p* = 0.002, df = 20, MS = 27, two-way ANOVA with Tukey HSD, Fig 4). The *Synechococcus* food resulted in a significantly lower MUFA content in *Daphnia-*HL body than *Nannochloropsis* food at each respective temperature (*p* = 0.0001 at 18°C and *p* = 0.0002 at 26°C, df = 20, MS = 27). Although the PUFA content in *Daphnia-*HL body was lower in *Synechococcus* food and at elevated temperatures (Fig 4), only the combined effect of increased temperature and the low quality cyanobacterial food resulted in a significantly lower body PUFA content in 6-d old individuals (FQ × T, p = 0.007, df = 20, MS = 25, two-way ANOVA with Tukey HSD).

## Discussion

The high latitude *Daphnia* isolated in the present study (*Daphnia*-HL) was identified to be a triploid representative of the *D*. *pulex* species complex. This is consistent with previous observations that a large number of subarctic clones of this species are triploid while temperate clones, including the *Daphnia*-T used in our experiments, are diploid [26]. Our results showed that the *Daphnia*-HL feeding curve was strikingly different from that for *Daphnia*-T, with a much lower threshold food concentration (TFC) for growth. *Daphnia*-HL was negatively affected by subarctic picocyanobacteria as a food source, and this effect greatly worsened at the increased temperature. This implies that high latitude aquatic ecosystems may be affected to a greater extent by indirect trophic effects than by the direct physiological impacts of climate warming. It should be noted, however, that these results are for a single subarctic clone of *Daphnia pulex*, and whether they are general features of high latitude *Daphnia* will require more detailed studies on a wide range of species and clones.

### Clonal origin effects

The growth rate—food curves for both *Daphnia* clones closely fitted the expected relationship, but with major differences in the curve parameters, especially the TFC values. *Daphnia*-T at 18°C had a TFC of 17.5 μg C L^-1^, which is similar to that of other temperate *Daphnia pulex* clones fed nutrient replete phytoplankton (e.g., 20 μg C L^-1^, using larger phytoplankton cells, [21]). In contrast, *Daphnia*-HL maintained positive growth rates above a threshold of 8.1 μg C L^-1^. This TFC value was half that of the temperate clone and well below most values reported in the literature. This pronounced difference implies that the high latitude daphnids may be well adapted to low food concentrations, which is a feature of oligotrophic northern lake and pond environments [17]. The slope of the growth-food curves for the two clones also differed significantly, with the slope for *Daphnia*-HL 43% below that for *Daphnia*-T. This muted response to increases in food concentration by the high latitude clone might also be a reflection of the persistently low phytoplankton biomass concentrations in northern waters.

### Temperature effects

The distinct growth-food characteristics of *Daphnia*-HL implied that this clone is well attuned to cold subarctic environment, hence we hypothesized that it would be more negatively affected by warming than the temperate counterparts. The 8°C increase in temperature indeed resulted in a significant increase in TFC, but there was no significant change in slope. The temperate clone showed both a significant increase in TFC and a significant decline in slope. An increase in TFC values as a function of temperature has been previously reported in zooplankton and suggested as an explanation for the dominance of small cladocerans in tropical climates [34]. However, in an experimental comparison of several cladocerans, although TFC values were found to increase with increasing temperature, there was no significant effect of body size [35]. Our results lend further support to the view that high latitude zooplankton have a number of distinctive features, including better competitive ability at low food concentrations [36].

### Polyploidy effects

Many hypotheses have been proposed to account for the shift in crustacean zooplankton towards polyploidy at high latitudes. Proximal hypotheses invoke higher rates of unreduced gamete production at lower temperatures [37] or hybridization between refugial species following Pleistocene glaciations [38]. Functional hypotheses include greater flexibility of polyploid organisms under a suite of environmental conditions [39] and better competitive abilities of polyploid organisms against inbred sexual populations in marginal habitats [40]. The results obtained here provide some support for the functional hypotheses, in that *Daphnia*-HL was competitive at low food concentrations. However, the growth and feeding performance of *Daphnia pulex* is known to be variable among clones [41], and additional experiments are required to assess the interclonal variability in temperature and food effects on northern and southern communities.

### Food type effects

There was a clear difference in food quality between the two picophytoplankton food types, specifically in total fatty acid content and in presence or absence of PUFAs. Consistent with this difference in food quality, *Daphnia*-HL showed a strong negative response to the picocyanobacterial food supply, with a pronounced increase in its TFC. The nutritional status of the animals was also impaired by their feeding on picocyanobacteria, as shown by the decreased total fatty acid content in all lipid classes. The inadequacy of cyanobacterial food for zooplankton is well known [42,43], with effects that include lower fecundity, survival and growth, and a decline in the size structure of the population [44]. Our results showed that cyanobacteria not only strongly affected the feeding threshold, but also had a significant effect on the slope of the growth rate versus food abundance curves. This combined change in both TFC and slope may imply compensatory feeding [45], and such a response by *Daphnia*-HL encountering picocyanobacteria could have other indirect effects, for example an increased ingestion and bioaccumulation of cyanotoxins and toxic metals in a warmer climate [46,47].

### Interactions between temperature and food type

The possibility of interactions between temperature and food quality on zooplankton feeding efficiency was first suggested by Cole *et al*. [48] and has been increasingly recognized as a potentially important response to environmental change [49,50]. In the present study, the temperature increase did not result in a significant change in slope of the growth-food curve for *Daphnia*-HL, but the combined effect of warming and picocyanobacterial food resulted in a significant change of slope and in a greater than 6-fold increase of the TFC. This result implies a synergistic rather than additive effect of these two factors on high latitude zooplankton competitiveness, and indicates a potentially greater importance of indirect relative to direct effects of climate warming. The indirect effects of temperature change also included the decrease in polyunsaturated fatty acid content, which in turn may have led to lower survival, fecundity and growth rates. Schlechtriem *et al*. [51] showed that daphnids cultured at elevated temperatures and fed cyanobacteria food suffered from decreased fatty acid content and composition, which shifted to that reflecting their food [52]. Our study support these findings in *Daphnia*-HL and also points to preferential retention or biosynthesis of unsaturated fatty acids in this zooplankton strain [53]. However, temperature changes that stimulate cyanobacterial development in high latitude lakes and ponds [12] may also alter the composition and dynamics of fatty acids in picocyanobacterial food [54,55]. The indirect effects of global warming on high latitude zooplankton communities may thus be stronger than indicated by our results, and climate-zooplankton models of the direct effects of temperature and food quantity (e.g., [56]) could be usefully modified to include a food quality component.

### Implications for subarctic aquatic ecosystems in a warming climate

Global climate change is likely to strongly alter the community structure of high latitude aquatic ecosystems [1]. Higher air temperatures and accelerated permafrost thawing will likely result in greater nutrient and organic carbon loading to high latitude lakes and ponds. Cyanobacteria are already a common element of the phytoplankton in many northern lakes and rivers [9,11,12], and this combination of warming and enrichment may stimulate their development and dominance in a manner similar to that observed in temperate climates [57]. Although the present study was limited to one clone of high latitude *Daphnia* and one strain of high latitude cyanobacteria, the pronounced nature of the responses implies that these effects deserve close attention.

*Daphnia* has been used for many decades as a model organism to study the effects of environmental stressors on zooplankton in various aquatic ecosystems [58], and it plays a key trophic role in the food webs of arctic lakes and ponds [17]. It has been suggested that *Daphnia* populations will further expand in high latitude lakes as a result of climate-induced increases in lake productivity [46]. However this effect may be offset by a phytoplankton shift towards cyanobacteria, and a synergistic negative effect of lesser food quality and warming on *Daphnia* feeding and growth. Such effects would impair the transfer of carbon to high trophic levels, and may reduce the impact of grazing on cyanobacteria, further contributing to their growth and dominance. As shown here, these indirect impacts of temperature change on phytoplankton-zooplankton interactions may greatly exceed the direct positive effects of temperature on zooplankton growth and physiology.

## Supporting Information

S1 Dataset Excel spreadsheet containing detailed information on *Daphnia* growth rates, FA content and composition, *Daphnia* neonate mass and phytoplankton carbon content calculation. (XLS)

S1 Fig Maximum likelihood of phylogenetic relationships among members of *D*. *pulex* complex using partial sequences of the ND5. The tree is rooted through the European *D*. *pulex* group. Maximum likelihood bootstrap values for PhyML are indicated for major groups. Arrows show the position of the two *Daphnia* clones used in this study.
(PDF)
