# Supplementary figures and images for: Climate Effects on High Latitude Daphnia via Food Quality and Thresholds
Source: PLoS One. 2015 May 13;10(5):e0126231. doi: 10.1371/journal.pone.0126231 (PMC4430472; doi:10.1371/journal.pone.0126231)

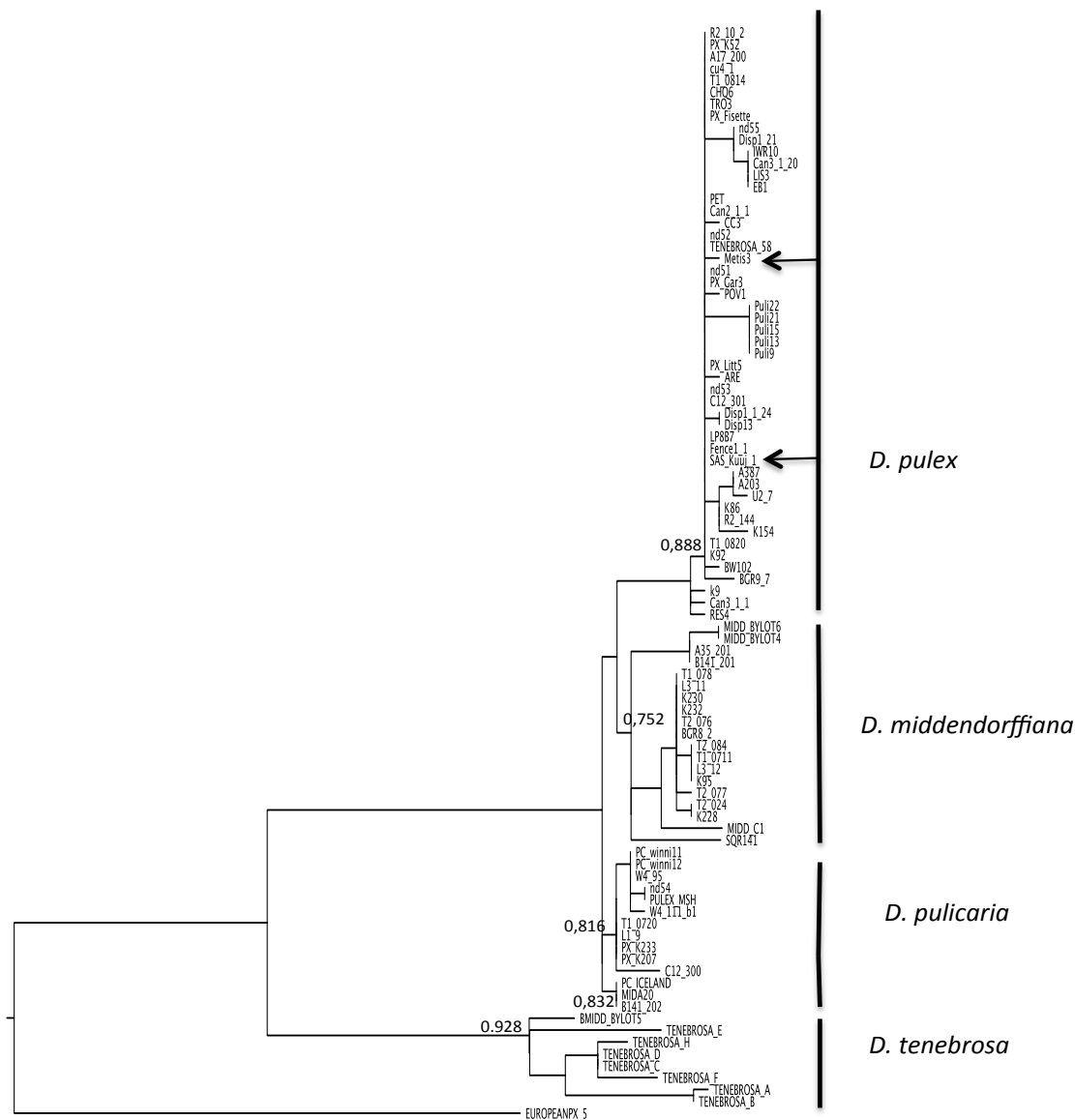

Supplement: S1 Fig — The tree is rooted through the European D. pulex group. Maximum likelihood bootstrap values for PhyML are indicated for major groups. Arrows show the position of the two Daphnia clones used in this study. (PDF) [file pone.0126231.s002.pdf]
